# Is It Worth Considering Colonic Evaluation After Appendicectomy?

**DOI:** 10.7759/cureus.43248

**Published:** 2023-08-09

**Authors:** Ramprasad P Rajebhosale, Nathan M Robinson, Nayaab A Kader, Iyomi Chathurika Ratnayake, Mitalee H Sawant, Vijitha Chandima Halahakoon

**Affiliations:** 1 General and Colorectal Surgery, Colchester General Hospital, East Suffolk and North Essex NHS Foundation Trust, Colchester, GBR; 2 General Surgery, Colchester General Hospital, East Suffolk and North Essex NHS Foundation Trust, Colchester, GBR

**Keywords:** caecal polyp, cecal mass, colonoscopy, colorectal cancer, appendicectomy

## Abstract

Introduction

The association of acute appendicitis with caecal or colorectal cancer is known. One of the proposed theories for acute appendicitis is luminal blockage by mass at the base of the appendix. There have been no national recommendations or guidelines for follow-up with patients aged 40 and older after an emergency appendicectomy. The purpose of this study was to evaluate the prevalence of caecal and colonic cancer or polyps in patients over the age of 40 who have undergone an appendicectomy. This shall enable us to develop the necessary strategies to investigate and diagnose associated caecal and colonic pathology in acute appendicitis to prevent delayed diagnosis of colon cancer.

Methods

All patients who underwent appendicectomy between October 2011 and October 31, 2021, and who were 40 years of age or older were included in this retrospective cohort study. Patients aged 40 to 54 years old and patients 55 years or older underwent subgroup analyses. We looked at any investigations of the colon (CT pneumocolon or colonoscopy) within three years before the appendicectomy or three years after an appendicectomy. All colorectal cancers diagnosed within five years of the index episode of appendicitis were included in the analysis.

Results

A total of 1076 appendicectomies were performed on patients aged 40 and older during the study period of 10 years. A total of 769 patients were confirmed to have appendicitis on histology. One hundred and fifty-seven patients had colonic investigations within three years of the diagnosis of acute appendicitis. In our study, 51 of the 769 patients (6.63%) were found to have colorectal neoplasms. Eight patients (8/769, 1.04%) were diagnosed with colorectal cancers, and the occurrence of caecal cancer was 0.26% (2/769). The mortality rate was 75% (6/8) in these patients diagnosed with colorectal cancer. Four out of six died due to advanced metastatic colonic cancer. In comparison to patients aged 40 to 54, patients over the age of 55 had a statistically significant increased risk of caecal pathology (polyp and cancer) (p = 0.07).

Conclusion

There seems to be an increased risk of significant colorectal neoplasm in patients over the age of 55 who are admitted with acute appendicitis, and there appears to be an increased severity with a poor prognosis of cancer in these individuals. We recommend the use of routine colonoscopy or CT pneumocolon, particularly for those over the age of 55 who present with acute appendicitis or the histology of appendicular neoplasms.

## Introduction

Appendicectomy for acute appendicitis is one of the most frequently performed surgical operations. One of the proposed theories for acute appendicitis is luminal blockage by faecolith, lymphoid hyperplasia, or mass at the base of the appendix [1]. The association of right-sided colon cancer with acute appendicitis was first described by Shears in 1906, and this has become a well-recognized and well-reported entity [2]. The incidence of acute appendicitis as the presenting symptom of caecal or ascending colon cancer is reported to be 3.4% to 15% [3]. It is not unusual to miss the primary cause of an inflamed appendix. Mayo reported in a retrospective analysis from 1941 that 15% of 885 individuals with caecal and ascending colon tumors exhibited symptoms consistent with carcinoma but had undergone an appendicectomy before the cancer was diagnosed [4]. When appendicitis and carcinoma of the caecum coexist, the symptomatology of appendicitis dominates the picture. Anemia, weight loss, and changes in bowel habits would favor carcinoma, but such symptoms are by no means always present.

Although the occurrence of appendicitis with caecal cancer is uncommon, it should be considered in patients over the age of 40 [5]. Various studies in the literature documented the increase in the incidence of colonic cancer with advancing age coexisting with appendicitis [6,7]. In the past, there has been great improvement in the diagnosis of colorectal cancers with computed tomography and colonoscopy compared with the traditional lower midline laparotomy to feel the caecal base in acute appendicitis in elderly patients [8]. Out of the various modalities available for the diagnosis of colorectal cancer, colonoscopy is considered the gold standard. This also allows for confirming the diagnosis with histology.

Although there has been a proven association between acute appendicitis and right-sided colonic cancers and the incidence of colorectal cancer in patients presenting with acute appendicitis is higher than in the general population over 40 years of age, there have been no national recommendations or guidelines for the follow-up of these patients. This study aimed to assess the incidence of significant caecal and colonic pathologies in patients over 40 who have undergone appendicectomy. This will enable us to develop the necessary strategies to investigate and diagnose associated caecal and colonic pathology in acute appendicitis to prevent delayed diagnosis of colon cancer.

## Materials and methods

Methods

This retrospective cohort study collected data for all patients aged 40 years or older who underwent appendicectomy during the period of 10 years between October 2011 and October 31, 2021, at Colchester Hospital, East Suffolk and North Essex Foundation NHS Trust. Patients were identified using the hospital’s electronic database. Only emergency appendicectomies were included in this retrospective study, and those patients who had undergone appendicectomy as a part of a more extended procedure were excluded (e.g., right hemicolectomy). All patients undergoing appendicectomy had their histology results examined. This study included patients who had undergone appendicectomy as the index procedure and had been diagnosed with acute appendicitis based on histology for additional analysis.

Those aged 40 years or older were chosen since patients in this age range are frequently mentioned in published data. Patients between 40 and 54 years old and patients 55 years of age or older underwent subgroup analyses. Comparing "younger" and "older" patients allowed for a better understanding of any differences that might exist in colorectal cancer development in both groups. Patients with normal appendicular histology (excluding appendicitis) were excluded from the main analysis. The hospital's audit department approved this project (approval no. GSA996).

Data

The data gathered encompassed demographic information, the date of appendicectomy, preoperative CT imaging at the initial admission, and histological diagnosis. We also looked at any investigations of the colon (CT pneumocolon or colonoscopy) within three years before the appendicectomy or within three years after the appendicectomy. A comparison of the rates of colorectal pathologies (polyps and cancer) was made between patients aged 40 to 54 years and 55 years and older. Patients with colorectal pathologies were identified by reviewing electronic reports for imaging and endoscopic procedures. We also cross-referenced the hospital’s colorectal cancer database to identify: any colorectal cancers in patients who did not have any colonic investigations three years before or after their index admission with histologically proven appendicitis; any colorectal cancers in patients who were found to have colorectal polyps in their colonic investigations. All colorectal cancers diagnosed within five years of the index episode of appendicitis were included in the analysis as related to the episode with appendicitis, especially as most colorectal neoplasms will take about five years to evolve [9].

Statistical analysis

Descriptive analytics were employed to characterize the data. Odds ratios were calculated to compare age groups 40 to 54 years and 55 years and older for any significant difference between them when they were found to have caecal pathologies associated with appendicitis. Based on previous studies, the association of colorectal cancer with acute appendicitis in patients aged 40 and older is 2.4%, and the incidence of colorectal cancer in patients aged 40 and older in the UK in the general population is 0.27%. Considering the probability of a type I error of 0.05 and the power of the study, 90% of the sample size necessary was 196 [10].

## Results

A total of 1076 appendicectomies were performed in the study population of 40 and older during the study period of 10 years. A total of 78% of operations were performed for acute presentations of abdominal pain. Around 91.43% (769/841) were confirmed cases of acute appendicitis on histopathological reports (Table 1). The negative appendicectomy rate was 8.56%. A total of 769 patients with confirmed appendicitis on histology were included in the study cohort for subsequent analysis. The median age of the participants was 56 years, ranging from 40 to 93 years. A total of 52% (399/769) of patients were aged 40 to 54 years, and 48% (370/769) of patients were aged 55 years or over.

**Table 1:** Appendicectomies

Findings	N	Percentage
Total appendicectomies	1076	100
Emergency appendicectomies	841	78.16
Histologically confirmed acute appendicitis	769	91.43

Of all the patients who had an appendicectomy, 80.5% had either a CT scan on admission, a colonoscopy, or a CT pneumocolon within the preceding three years or three years after the appendicectomy (Table 2). Some of the patients had combinations of more than one investigation. Only 20% (157/769) of patients had some form of colonic evaluation in the form of colonoscopy or CT pneumocolon.

**Table 2:** Investigations

Investigation	N	Percentage
Colonoscopy	151	19.6
CT pneumocolon	6	0.8
CT Scan on admission	462	60
Total	619	80.5

A total of 157 patients had colonic investigations (colonoscopy and CT pneumocolon) within three years of the diagnosis of acute appendicitis (as seen in Table 2). A total of 46 patients had colorectal polyps. Out of these, 22 polyps were in the right colon. The findings of the colonoscopy evaluation are depicted in Tables 3-4. Fourteen cases were histologically confirmed as appendicular cancers after an emergency appendicectomy. Eight colonic cancers were noted in the study. Six of these eight patients had a colonic evaluation within three years of the diagnosis of acute appendicitis. Two patients were investigated after three years of index appendicectomy (Table 5). Both of these were diagnosed within five years. Both caecal cancers, one obstructing transverse colon cancer and one obstructing sigmoid cancer, could be attributed as likely causative factors responsible for acute appendicitis.

**Table 3:** Findings on colonoscopy evaluation (within three years before and after appendicectomy)

Findings		N (total N=157)	Percentage
Right colon	Polyp	22	14.0
	Cancer	2	1.27
Total colorectal	Polyp	46	29.3
	Cancer	6	3.81

**Table 4:** Findings on colonoscopy as per age-specific subgroup analysis IBD: Inflammatory bowel disease, DD: Diverticular disease

Pathology	Age 40 to 54 (N=399)	Age >55 (N=370)
Caecal polyp	2	8
Caecal cancer	1	1
Ascending colon polyp	0	2
Ascending colon cancer	0	0
Transverse colon polyp	0	1
Transverse colon cancer	0	1
Descending colon polyp	0	3
Descending colon cancer	0	0
Sigmoid polyp	2	7
sigmoid cancer	0	1
Rectal polyp	1	10
Rectal cancer	0	2
Others (IBD, DD, etc.)	17	37
Polyps (multiple sites)	1	9

**Table 5:** Total colorectal cancers

Cancer site	Colonic evaluation (N=157)	No colonic evaluation (N=612)
Caecum	2	0
Transverse colon	1	1
Sigmoid colon	1	1
Rectum	2	0

In total, eight cases were diagnosed with colorectal cancer after emergency acute appendicectomies. As such, the overall incidence of colon cancer in our study was 1.04% (8/769), and the occurrence of caecal cancer was 0.26% (2/769). The mortality rate was 75% (6/8) in patients diagnosed with colorectal cancer. Four out of six died due to advanced metastatic colonic cancer. The demographic characteristics of the patients who had cancer are depicted in Table 6.

**Table 6:** Demographics of colorectal cancer cases PET: Positron emission tomography, IDA: Iron deficiency anaemia

Characteristics	Colonoscopy						No colonoscopy within three years	
Age	51	61	68	73	76	81	63	78
Sex	M	M	F	F	M	M	M	M
Findings	Caecal cancer at the base of the appendix	Obstructing hepatic flexure cancer	Rectal cancer	Rectal cancer	Caecal cancer at the base of the appendix	Obstructing sigmoid cancer (2014)	Sigmoid polypoidal cancer (2017)	Transverse colon cancer (2018)
Histology	Dukes C1, T3N2bMx	Dukes C2, T4N2	Dukes A, T2N0	Dukes A, T1N0	Dukes B, T4N0			Dukes C1, T3N1
Outcome	Died two years after cancer resection surgery with advanced metastatic disease	Died 10 months after appendicectomy due to advanced metastatic cancer	Alive	Died three years post anterior resection due to advanced metastatic cancer	Died three years post right hemicolectomy due to advanced metastatic cancer	Died six years after surgery due to respiratory failure unrelated to colon cancer	Died three years post laparoscopic Hartmann’s and reversal later due to cardiac co-morbidities and aortic stenosis	Alive
Reason for colonoscopy	Arranged as a follow-up as the patient was anaemic and had a history of PR bleeding	Abnormal CT scan	PET scan spot for lymphoma follow-up investigation	Arranged as a follow-up post appendicectomy	CT suggestive of abnormality in the caecal region		Surveillance	Abdominal pain and IDA

Findings in participants less than 55 years of age

In patients younger than 55 years, the incidence of caecal pathology (polyps and cancer) was 0.75% (3/399). One patient was diagnosed with caecal cancer (0.25%, 1/399) and two with caecal polyps (0.5%, 2/399). Overall, in the colon and rectum, six polyps were noted (1.5%, 6/399) with two patients having caecal, two patients having sigmoid cancer, one patient having a rectal polyp, and a single patient having multiple polyps at different sites (as seen in Table 4).

Findings in participants older than 55 years of age

In patients aged more than 55 years, the incidence of caecal pathology (polyps and cancer) was 2.43% (9/370), and the incidence of caecal cancer was 0.27% (1/370). The latter patient had caecal adenocarcinoma. Eight patients had benign caecal polyps. None of the patients with polyps develop cancer later on. Overall, seven colorectal cancers (1.89%, 7/370) were noted in this age group: one in the caecum and two cancers in the transverse colon, sigmoid, and rectum, respectively.

Patients aged 55 years or older were found to be significantly more likely to possess caecal pathology (polyps and cancer) than those aged 40 to 54 (p = 0.07). The odds ratio of developing caecal pathology (polyps and cancer) was 3.3 times higher (95% CI 0.88-12.18) in individuals aged 55 years and above compared to people between the ages of 40 and 54 years. Patients over 55 were also found to be more likely to have associated colorectal cancer compared to those aged between 40 and 54, with an odds ratio of 4.35 (p = 0.19).

## Discussion

In our study, 51 of the 769 patients (6.63%) were found to have colorectal neoplasms after confirmed appendicitis in patients over the age of 40, and eight patients (8/769, 1.04%) were diagnosed with colorectal cancers. In the United Kingdom, the age-wise incidence of colorectal cancer in the same age group (40 and over) is 0.25% [11].

Lai et al. specifically examined individuals aged 40 and over, discovering that 0.85% of them had colorectal cancer at the time of appendicectomy or within 40 months of the initial operation [6]. They also stated that the overall incidence of colorectal cancer in patients over the age of 40 at any time after a diagnosis of appendicitis was 1.76%, and amongst these, 43% were caecal cancers [6]. Another study by Bizer et al. reported that 1.8% of patients over the age of 65 with proven appendicitis had underlying caecal cancer [7]. It has been noted in some studies that there is a sharp rise in the prevalence of colonic polyps over the age of 40 in the general population [12]. In the UK, the incidence of colon cancer in the general population of ages 40 to 45 is 0.006% [9]. Age-specific incidence rates rise steeply from around age 50 to 54, and in those above 55 years, it is 0.03% [13]. Moreover, the overall incidence of colorectal cancer is increasing in younger patients (aged <50 years) [14]. As such, surgeons should entertain the possibility of caecal pathologies in patients after middle age with appendicitis. In a recent retrospective study by Pedersen et al. in Norway, the distribution of colorectal neoplasms (cancer and adenomas) in patients aged 40 and over within three years of admission for acute appendicitis was detected in 54 of the 731 patients (7.4%) [15]. Nine patients (1.2%) were found to have colorectal cancer. A study by Shine et al. reported that patients ≥45 years who have had an appendicectomy had a 6.3-fold (CI 3.6-10.2) increased risk of colorectal carcinoma compared to the general population. Those patients aged between 45 and 60 years had a 17-fold increased standardized risk ratio. However, this study included not only caecal but colonic as well as rectal cancers [16]. The population study in Taiwan by Wu et al. demonstrated an overall hazard ratio of 14.7 (99.9% CI 8.66-2.50) for developing colorectal cancer after emergency appendicectomy across all ages [17]. Lai et al. also presented a similar pattern of incidence of colorectal cancer after emergency appendicectomy in patients older than 40 years, with a 38.5-fold increase in the odds ratio [6].

In our study of patients aged more than 55 years, the reported incidence of caecal pathology (polyps and cancer) was 2.43% (9/370) and the incidence of caecal cancer was 0.27% (1/370). The association of appendicitis with caecal cancer has been documented in recent studies. A study of patients aged 55 years or older undergoing appendicectomy for acute appendicitis by Mohamed et al. [5] reported that a total of 2.2% had some form of caecal pathology. Around 1.6% (7/452) of these patients were diagnosed with caecal cancer. In the literature, various other studies also report this association of appendicitis with caecal cancer: Lai et al. report it to be 0.85% [6], Bizer et al. at 1.8% [7], Khan et al. at 1.25% [18], and Pedersen et al. at 1.5% [15] of patients following appendicectomy (Table 7).

**Table 7:** Association of colorectal pathologies and appendicitis

Age group	Authors	40 to 54 years	>55 years	Country
Caecal polyp %	Mohamed et al. [5]	0.3	3	United Kingdom
	Pedersen et al. [15]	6.1		Norway
	Our study	0.5	2.16	
Caecal cancer %	Mohamed et al. [5]	0.7	1.6	United Kingdom
	Pedersen et al. [15]	0.96		Norway
	Lai et al. [6]	0.77		Taiwan
	Bizer et al. [7]	NA	1.8	United States
	Our study	0.25	0.27	
Colorectal cancer %	Cancer Research UK [11]	0.02	0.25	
	Shine et al. [16]	2.4		New Zealand
	Our study	0.25 (1/399)	1.89 (7/370)	

In the present study, cancers were evenly distributed across the colon, with two patients having caecal, transverse, sigmoid, and rectal cancers each. In the literature, some studies have noticed an uneven distribution of cancers. In a study by Pedersen et al. [15], seven of nine cancers were present in the caecum, thus favoring the hypothesis that there may be an association between appendicitis and right-sided colon cancers. In contrast, in a study by Bretthauer et al. [19], the distribution of proximal and distal cancers was about 20% and 80%, respectively (proximal colon is defined as from caecum to splenic flexure).

The prognosis of tumours occurring in the caecum or proximal colon presenting as acute appendicitis is reported as poor, with the reason partly being delayed diagnosis. In our study, six of eight patients died. Four of them died within three years of the diagnosis of colon cancer due to advanced metastatic disease, thus indicating a poor prognosis for patients presenting with acute appendicitis and having underlying colonic cancer.

Although appendicular cancers are rare, synchronous colorectal neoplasia has been reported in 3% to 5% of patients with appendicular neoplasia [20]. Consequently, if histological examination of the appendix reveals any such pathologies, these patients should receive a colonic evaluation. In our study, 14/769 (1.66%) patients who presented with acute appendicitis had appendicular cancer. Only 57% (8/14) of these had a colonoscopy within three years before or after the diagnosis of cancer. 

Older patients presenting with symptoms of acute appendicitis are often investigated with CT scans. Although patients can be reassured of no pathology on a negative initial CT scan, it has been shown in various studies that the accuracy of picking up caecal pathology in acute appendicitis remains low. Hence, post-appendicectomy colonoscopy is recommended in the elderly population. In our study, 468 (61%) patients were investigated with a CT scan. Only three CT scans raised the possibility of caecal cancer, of which none were found to have colorectal cancers. However, three patients presenting with acute appendicitis who were diagnosed with colon cancers on colonoscopies later were not picked up by the initial CT scan. In a study by Khan et al., of the 80 patients presenting with acute appendicitis investigated radiologically, a CT scan identified only 1 patient (1.25%) with suspicion of a caecal tumour [18]. In another study by Pedersen et al., the sensitivity of CT for discovering cancer was just 0.25 and the specificity was 0.97 with positive and negative predictive values of 0.12 and 0.99, respectively. Thus, we cannot rely on CT scans, and these patients warrant a colonic evaluation in the form of a colonoscopy or CT pneumocolon.

There are certain limitations to our study. The retrospective design of the study itself is an important limitation. Only 157 patients had a colonoscopy within three years of surgery for acute appendicitis, thus indicating a selection bias in requesting colonic investigations. With the set limit for the observation period at three years, those patients admitted in late 2019 and 2020 have not yet had a three-year observation period at the time of the study, and any findings in these patients after 30 March 2022 have not been registered. We did not include patients who were managed conservatively. The study could not explain the association of colorectal carcinoma with appendicitis, although the relation is explicable in caecal pathology. The study population has not undergone colonoscopy; hence, the actual incidence of colorectal pathology cannot be assessed. Finally, we may have missed those patients who were investigated elsewhere.

## Conclusions

There seems to be an increased risk of significant colorectal neoplasm in patients over the age of 55 admitted with acute appendicitis, and there seems to be an increased severity and poor prognosis of cancer in these individuals. The sensitivity of CT scans to detect colorectal cancer in the setting of acute appendicitis is low. We recommend the use of routine colonoscopy or CT pneumocolon, particularly in those over the age of 55 years presenting with acute appendicitis or with histology of appendicular neoplasms. We also recommend that clinicians err on the side of caution in patients between the age of 45 and 54 years with appendicitis. Further randomized trials are necessary to increase the level of evidence.
